# The Social Costs of Ubiquitous Information: Consuming Information on Mobile Phones Is Associated with Lower Trust

**DOI:** 10.1371/journal.pone.0162130

**Published:** 2016-09-08

**Authors:** Kostadin Kushlev, Jason D. E. Proulx

**Affiliations:** 1 Department of Psychology, University of Virginia, Charlottesville, Virginia, United States of America; 2 Department of Psychology, University of British Columbia, Vancouver, British Columbia, Canada; Technion Israel Institute of Technology, ISRAEL

## Abstract

In an age already saturated with information, the ongoing revolution in mobile computing has expanded the realm of immediate information access far beyond our homes and offices. In addition to changing *where* people can access information, mobile computing has changed *what* information people access—from finding specific directions to a restaurant to exploring nearby businesses when on the go. Does this ability to instantly gratify our information needs anytime and anywhere have any bearing on how much we trust those around us—from neighbors to strangers? Using data from a large nationally representative survey (World Values Survey: Wave 6), we found that the more people relied on their mobile phones for information, the less they trusted strangers, neighbors and people from other religions and nationalities. In contrast, obtaining information through any other method—including TV, radio, newspapers, and even the Internet more broadly—predicted higher trust in those groups. Mobile information had no bearing on how much people trusted close others, such as their family. Although causality cannot be inferred, these findings provide an intriguing first glimpse into the possible unforeseen costs of convenient information access for the social lubricant of society—our sense of trust in one another.

## Introduction

Ever since the advent of the printing press in the 15^th^ century, humanity has been exposed to more information than a single person can consume in a lifetime [1]. But the recent development of digital technology and the Internet has ushered us in a whole new age of information [2]—one in which the wealth of human knowledge is but a mouse click away. By 2007, Americans were consuming five times more information than they did in the 1980’s [3]. But until 2007, this information consumption was largely constrained to people’s homes and desks. As Steve Jobs promised in 2007, however, smartphones have ‘changed’ everything by expanding the realm of instant information far beyond our homes and desks. These mobile computing devices provide unprecedented access to information, enabling individuals to satisfy their information needs virtually anytime and anywhere.

Mobile computing has changed *where* people access information, and it has also changed *what* information people seek. Like previous information media—from newspapers to TV and radio—mobile phones are used to keep up to date with news and current affairs [4]. But more frequently than that, people use their mobile phones to access new types of information—from finding out about current social events to finding information about how to get there [4]. Using a nationally representative sample of Americans from the latest wave (Wave 6) of the World Values Survey, we were able to explore whether these recent changes in information access have any bearing on the social lubricant of society—people’s sense of trust in one another.

Across disciplines, from sociology and psychology to economics and medicine, trust [5] is viewed as critical for individual well-being, economic prosperity, and even physical health [6–12]. Trust has been theorized to arise from people’s interdependence with one another [13]. May depending on technology affect how much people depend on one other? One possibility is that by changing where and what information people have access to, ubiquitous computing may decrease people’s interdependence with other members of society—especially those we normally encounter only outside of the sphere of close relationships. Only a decade ago, a person lost in an unfamiliar city might have had to rely on the kindness of strangers to find her way. Today, finding directions is only a finger swipe away on the map app on one’s phone, obviating the need to rely on friendly strangers. And by forgoing casual social interactions, people may also be forgoing opportunities to cultivate the weak social bonds that hold society together. Indeed, recent research has shown that even brief casual social interactions with strangers and acquaintances can foster our a sense of connection with others [14,15]. From this perspective, relying on mobile phones for information may be uniquely associated with lower trust in people outside of our close social circles of friends and family.

To explore the relationship between mobile information and trust, we analyzed data from a large nationally representative sample of Americans in the most recent wave (Wave 6) of the World Values Survey. Respondents were asked to indicate how frequently they relied on their mobile phones and on other sources for obtaining information, as well as how much they trusted people of different groups—from family to neighbors and strangers. Thus, we were able to explore the relationship between mobile information and trust and contrast this relationship with how using other media for information relates to trust. Furthermore, we examined whether the relationship between relying on phones for information and trust depends on the strength of the social bond—from distant others (e.g., strangers) to close others (e.g., family).

## Materials and Methods

We used data from Wave 6 of the World Values Survey (WVS) of 2232 respondents (*Median age* = 46; 52% female); Out of these respondents, 45 did not respond to the question about how frequently they used their mobile phones for information, leaving 2187 participants for analyses on this critical variable (*Median age* = 46; 52% female). We applied the weights provided by WVS, thus adjusting the sample to represent the US population. We did not seek approval from our institutional ethics board because our institutional ethics board considers the use of publicly-available data exempt. All participants underwent the appropriate consent procedures under the World Values Survey.

Respondents were asked to indicate how frequently they relied on various sources to obtain information (1 –*daily*, 2 –*weekly*, 3 –*monthly*, 4 –*less than monthly*, 5 –*never*). We recoded the scales so that higher scores indicated greater frequency of obtaining information through each source. In addition to mobile phones, the sources included daily newspapers, printed magazines, TV news, radio news, email, the Internet, and familiar others/colleagues. In order to form a composite score indicating people’s overall tendency to seek information using methods other than mobile phones, we calculated the average of all methods of obtaining information except mobile phones (*α* = .72).

Participants were also asked to indicate how much they trusted people of various groups: 1 –*completely*, 2 –*somewhat*, 3 –*not very much*, 4 –*not at all*. We recoded the scales so that higher scores indicated higher trust in people of each group. Specifically, people were asked how much they trusted their family, neighborhood, people they knew personally, people they were meeting for the first time, people of other religions, and people of other nationalities. For convenience in describing the findings, we use *strangers* in lieu of ‘people you met for the first time’, and *neighbors* in lieu of ‘neighborhood’.

Respondents were also asked to report their age, sex (*male*, *female*), race/ethnicity (e.g., *White*, *Black*, *East Asian*, *Hispanic*), and employment status (e.g., *full-time*, *part-time*, *retired*, *student*). They also reported their highest level of education on a scale from 1 –*no formal education* to 9 –*university degree*. In addition, respondents estimated what income group their family belonged to within their country on a scale from 1 *–lowest group* to 10 *–highest group*. Full information on all variables can be obtained from the WVS website: http://www.worldvaluessurvey.org/WVSDocumentationWV6.jsp.

## Results

The more people relied on their phones for information, the less they trusted neighbors, strangers, and people from other religions or nationalities (Table 1). Importantly, relying on mobile phones for information had no bearing on how much people trusted members of their own family or familiar others. This pattern of findings is consistent with the possibility that the access to mobile information may be eroding trust by compromising the building of weak bonds with other members of the community but not with close others.

**Table 1:** Regression analyses: Using mobile phones, but not other media to obtain information predicts distrust.

		Standardized Regression Coefficients (Betas)					
	Trust in…	Strangers	Neighbors	People from another religion	People from another nationality	Familiar others	Family
Freq. of using phones for information	With no controls^1^	−.07**	−.05*	−.08***	−.11***	−.02	.03
	Controlling for using other media^2^	−.15***	−.15***	−.18***	−.20***	−.11***	−.04
	Controlling for using other media & demographics^3^	−.08***	−.05*	−.10***	−.14***	−.07**	−.02
Freq. of using other media for information	With no controls^4^	.14***	.19***	.19***	.14***	.18***	.15***
	Controlling for using phones^2^	.20***	.24***	.26***	.22***	.22***	.16***
	Controlling for using phones & demographics^3^	.12***	.14***	.16***	.13***	.17***	.12***

*Notes*. All numbers are standardized regression coefficients corresponding to *b*_1_ or *b*_2_ from the equations provided below. Demographic factors include age, sex, income, education, employment status, race and ethnicity. Other media include daily newspapers, printed magazines, TV news, radio news, email, the Internet, and friends/colleagues; ratings for those sources of information were averaged to form an overall composite of obtaining information through methods other than phones.

^*^p < .05

^**^p < .01

^***^p < .001

Regression equations

^*1*^ Trust = *b*_0_ + *b*_1_ Info-on-phone + *e*

^*2*^ Trust = *b*_0_ + *b*_1_ Info-on-phone + *b*_2_ Info-on-other-media + *e*

^*3*^ Trust = *b*_0_ + *b*_1_ Info-on-phone + *b*_2_ Info-on-other-media + *b*_3_ Age + *b*_4_ Sex + *b*_5_ Income + *b*_6_ Education

+ *b*_7_ Full-time-employed + *b*_8_ Part-time-employed + *b*_9_ Self-employed + *b*_10_ Retired + *b*_11_ Housewife + *b*_12_ Student + *b*_13_ Unemployed + *b*_14_ White + *b*_15_Black + *b*_16_ South-Asian + *b*_17_ East-Asian + *b*_18_Arabic + *b*_19_Hispanic + *e*

^*4*^ Trust = *b*_0_ + b_1_ Info-on-other-media + *e;*

To the extent that information media covers primarily negative stories (e.g., war, terrorism, crime [16]), keeping informed of what is going on in the world may breed distrust regardless of how and where people access the information. In contrast to this possibility, we found that people who obtained information through media other than mobile phones—including radio, TV, newspapers, and even the Internet—trusted others more (Table 1; see Table 2 for a break-down of correlations by each media). Interestingly, people who obtained information through their phones were also more likely to seek information through other sources, suggesting that some people have a stronger tendency to consume information than others. But even after controlling for the average frequency of using sources other than phones to obtain information (*α* = .72), people who more frequently obtained information on their phones still trusted others less (Table 1). Demographic factors measured in the survey—age, sex, income, education, employment status, and race and ethnicity—could also not explain why people who frequently sought information on their mobile phones trusted others less (Table 1).

**Table 2:** Table of correlations and descriptive statistics.

	*Information Source*								*Trust*					
	Newspapers	Maga-zines	TV news	Radio news	Mobile phones	Email	Inter-net	Friends/ colleagues	Strangers	Neigh-bors	People of another religion	People of another nationality	Family	Familiar others
Mean (Standard Dev.)	3.27 (1.53)	2.51 (1.10)	4.17 (1.22)	3.53 (1.48)	2.58 (1.75)	3.32 (1.61)	3.89 (1.43)	4.02 (1.14)	2.20 (.71)	2.76 (.68)	2.71 (.68)	2.66 (.68)	3.65 (.61)	3.21 (.61)
Correlations (r)														
Newspapers	1	.478***	.385***	.227***	.022	.153**	.068**	.164***	.123***	.172***	.153***	.110***	.094***	.130***
Magazines		1	.249***	.274***	.202***	.272***	.251***	.274***	.117***	.158***	.124***	.121***	.083***	.107***
TV news			1	.291***	.044*	.114***	.064**	.227***	.056**	.159***	.129***	.081***	.130***	.097***
Radio news				1	.152***	.203***	.225***	.298***	.073***	.08***	.095***	.069**	.082***	.081***
Mobile phones					1	.500***	.386***	.340***	−.073***	−.050*	−.079***	−.113***	.025	−.024
Email						1	.678***	.447***	.071***	.079***	.098***	.058**	.062**	.096***
Internet							1	.455***	.051*	.045*	.065**	.051*	.080***	.091***
Friends/ colleagues								1	.126***	.147***	.169***	.141***	.130***	.201***
Trust in strangers									1	.467***	.509***	.502***	.195***	.409***
Trust in neighbors										1	.446***	.394***	.331***	.468***
Trust in people of another religion											1	.747**	.238**	.424**
Trust in people of another nationality												1	.229**	.418**
Trust in family													1	.380**
Trust in familiar others														1

* p < .05

** p < .01

*** p < .001

In addition to the demographic factors examined above, the type of region people live in—rural or urban—may be another factor that could explain why consuming information on mobile phones is associated with lower trust. People in rural areas, for example, may be less likely to use their phones for information while also being more likely to trust their neighbors. The World Values Survey, however, provides no data on whether respondents reside in an urban or rural area, precluding us from controlling for this factor on the individual level. The most specific geographical information provided in the survey is the state in which respondents lived at the time of the interview. Combining this geographical information with data from US Census Bureau (https://www.census.gov/geo/reference/ua/urban-rural-2010.html), we calculated the percent of people in each US state who live in urban areas and the percent of people who live in rural areas. Thus, for example, most people reside in urban areas in states such as New York (83%) and California (90%), but not in states such as West Virginia (33%) and Montana (26%). This between-state variation in the proportion of residents who live in urban versus rural areas allowed us to examine whether the type of residential area could explain the relationship between trust and using phones to obtain information.

To control for urban and rural population at the state level, we employed multilevel modeling (MLM), treating person as Level 1 and state as Level 2 in the analyses. Thus, in a series of MLM analyses, we predicted person-level trust in each group (e.g., strangers) from person-level frequency of using phones for information, while controlling for state-level proportion of the population living in urban areas and proportion living in rural areas. For each analysis, we estimated the fixed effects of both person-level and state-level predictors. Just as in a regular regression, if the urban/rural proportion of the population of the state of residence accounts for the relationship between trust and the frequency of using phones for information, we should find nonsignificant fixed effects of using phones for information on trust. For each model, we also estimated the random intercept, which accounts for clustering within state.

As shown in S1 Table, the results of the MLM analyses mirrored the findings of the regular regressions presented in Table 1. Thus, even after controlling for variation in the proportion of urban and rural residents between states, people who used phones for information more frequently were less likely to trust strangers (*p* = .001), neighbors (*p* = .035), people of other religions (*p* < .001), and people of other nationalities (*p* < .001). Using phones for information was not associated with trust in familiar others (*p* = .444) or in family (*p* = .295; see S1 Table for details). In sum, after accounting for a wide range of alternative explanations, we found that people who consumed information through their mobile phones trusted strangers, neighbors, and outgroup members less.

## Discussion

Throughout history, transformative technologies—from the mechanical watch to the automobile and TV—have generated concerns about unintended consequences on social and societal well-being [17,18]. While speculation about the social costs of mobile technology is rampant, we found empirical evidence that the convenience inherent in always-accessible information is associated with lower trust. In a large nationally representative sample of Americans, we found evidence that consuming information through mobile phones—but not through other common methods—predicted lower trust in other members of society. Specifically, using data from the latest wave of the World Values Survey (Wave 6), we found that people who relied more on their phones to obtain information trusted strangers less. In contrast, relying on phones for information had no bearing on how much people trusted close others, such their family.

We theorized that mobile information erodes trust in strangers by interfering with casual opportunities to talk with strangers and by obviating the need to rely on others. Although our findings were robust to controlling for a range of demographic factors and other possible confounds, the correlational nature of our findings does not allow us to account for all conceivable alternative explanations for our findings. Unlike TV, radio, or newspapers, for example, information consumed on mobile phones comes from the World Wide Web; and recent research suggests that the Internet is a prime medium for the distribution of misinformation and especially of conspiracy theories [19], which may increase distrust. People also use their mobile phones to access information through methods such as social media, which are unavailable in more traditional information media. If these new types of information people access on their phones could explain our findings, however, we would expect to find that consuming information on the Internet more broadly would also predict lower trust. But we found that accessing information on the Internet in general predicts higher trust. Consistent with our theorizing, therefore, this pattern of findings suggests that it is indeed the mobility of information access that is associated with lower trust. Still, the present findings should be seen only as laying the groundwork for future research to examine how, when, and why the current technological revolution in mobile computing may be eroding trust.

We would be remiss, of course, if we did not acknowledge the reverse causal possibility: People who trust others less might be more likely to use their mobile phones for information. If this is the case, we might expect that controlling for how much people rely on other people for information would attenuate the relationship between mobile information and trust. Yet, we found the opposite: Controlling for how much people relied on other people for information did not explain away the negative relationship between mobile information and trust. Still, the only way to establish a clear direction of causality is to experimentally induce people to either rely on their phones for information or not, and see whether this manipulation has consequences for trust. Of course, it would be challenging to run an experiment with a nationally representative sample while measuring people’s natural behavior. These limitations of possible experimental designs underscore the advantages of the current correlational data.

We found an effect exclusively of mobile information that is specific to trust in strangers and other outsiders. Such specificity of the effect makes our findings theoretically significant, opening tantalizing questions for further investigation. But are these findings practically significant? The effects of mobile information on trust were small. To find large effects on trust of a simple shift in how people consume information would, of course, be strange. But given the importance of trust for the functioning of society, even these small effects can have big practical implications. Trust has been shown, for example, to underlie optimal deal making in economic transactions [11]. Beyond the health of the economy, trust has also been implicated in the health and well-being of individuals [9,10]. Thus, as new technologies continue to revolutionize how millions of people access information around the globe, even tiny effects on trust can have big implications for the health of individuals, economies, and nations. To illustrate, consider the example of aspirin, which has a statistically tiny effect on reducing risk of heart attack, explaining as little as one tenth of one percent of the variance [20,21]. Yet, when prescribed to millions of people, such a statistically small effect can save thousands of lives—a practically large effect.

Organisms tend to seek the easiest way to achieve the greatest outcome [22]. This Principle of Least Effort has been identified as one of the main principles guiding information seeking behavior [23]. Just as information technology continues to make our lives easier, our findings highlight the possible unforeseen social costs of instant, ubiquitous information access: By turning to convenient electronic devices, people may be forgoing opportunities to foster trust—the social lubricant of society.

## Supporting Information

S1 Table Multilevel models: Relying on phones for information as a predictor of trust clustered within state of residence. (DOCX)
