# Supplementary material for: The Social Costs of Ubiquitous Information: Consuming Information on Mobile Phones Is Associated with Lower Trust
Source: PLoS One. 2016 Sep 8;11(9):e0162130. doi: 10.1371/journal.pone.0162130 (PMC5015986; doi:10.1371/journal.pone.0162130)
Supplement: S1 Table — (DOCX) [file pone.0162130.s001.docx]

**Table S1. Multilevel models: Relying on phones for information as a predictor of trust clustered within state of residence.**

| **Model 1: Trust in strangers** |  |  |  |  |  |
| --- | --- | --- | --- | --- | --- |
| *Fixed Effects* | *Estimate* | *Std. Error* | *Df* | *t* | *p* |
| Intercept (*γ_00_*) | 2.056 | .480 | 63.57 | 4.29 | < .001 |
| Information on phones (*γ_10_*) | -.028 | .008 | 2254.52 | -3.26 | .001 |
| % urban popul. in state (*γ_01_*) | -.009 | .005 | 61.05 | -1.78 | .080 |
| % rural popul. in state (*γ_02_*) | -.010 | .007 | 58.29 | -1.58 | .119 |
| *Random Effects*  Intercept (*u_0j_*) | *Estimate*  .012 | *Std. Error*  .006 |  | *Wald Z*  1.83 | *p*  .067 |
| Residual (*ε_ij_*) | .493 | .015 |  | 33.13 | < .001 |
| **Model 2: Trust in neighbors** |  |  |  |  |  |
| *Fixed Effects* | *Estimate* | *Std. Error* | *Df* | *t* | *p* |
| Intercept (*γ_00_*) | 1.497 | .446 | 70.68 | 3.36 | .001 |
| Information on phones (*γ_10_*) | -.017 | .008 | 2248.49 | -2.11 | .035 |
| % urban popul. in state (*γ_01_*) | -.009 | .005 | 67.86 | -1.94 | .057 |
| % rural popul. in state (*γ_02_*) | -.008 | .006 | 65.57 | -1.32 | .192 |
| *Random Effects*  Intercept (*u*_0j_) | *Estimate*  .009 | *Std. Error*  .005 |  | *Wald Z*  1.75 | *p*  .081 |
| Residual (*ε*_ij_) | .451 | .014 |  | 33.11 | < .001 |

**Table S2. continued**

| **Model 3: Trust in people from other religions** | | |  |  |  |
| --- | --- | --- | --- | --- | --- |
| *Fixed Effects* | *Estimate* | *Std. Error* | *df* | *t* | *p* |
| Intercept (*γ_00_*) | 2.281 | .437 | 90.08 | 5.22 | < .001 |
| Information on phones (*γ_10_*) | -.030 | .008 | 2247.99 | -3.65 | < .001 |
| % urban popul. in state (*γ_01_*) | -.001 | .004 | 86.55 | -.33 | .744 |
| % rural popul. in state (*γ_02_*) | .000 | .006 | 83.07 | .00 | .998 |
| *Random Effects*  Intercept (*u*_0j_) | *Estimate*  .007 | *Std. Error*  .004 |  | *Wald Z*  1.76 | *p*  .079 |
| Residual (*ε*_ij_) | .459 | .014 |  | 33.20 | < .001 |
| **Model 4: Trust in people from other nationalities** | | |  |  |  |
| *Fixed Effects* | *Estimate* | *Std. Error* | *df* | *t* | *p* |
| Intercept (*γ_00_*) | 2.241 | .453 | 76.28 | 4.94 | < .001 |
| Information on phones (*γ_10_*) | -.045 | .008 | 2241.21 | -5.52 | < .001 |
| % urban popul. in state (*γ_01_*) | -.002 | .005 | 73.27 | -.36 | .721 |
| % rural popul. in state (*γ_02_*) | -.006 | .007 | 71.00 | -.98 | .330 |
| *Random Effects*  Intercept (*u*_0j_) | *Estimate*  .010 | *Std. Error*  .005 |  | *Wald Z*  1.95 | *p*  .051 |
| Residual (*ε*_ij_) | .446 | .013 |  | 33.10 | < .001 |

**Table S2. continued**

| **Model 5: Trust in familiar others** | | |  |  |  |
| --- | --- | --- | --- | --- | --- |
| *Fixed Effects* | *Estimate* | *Std. Error* | *df* | *t* | *p* |
| Intercept (*γ_00_*) | 1.415 | .387 | 63.20 | 3.66 | < .001 |
| Information on phones (*γ_10_*) | -.006 | .007 | 2247.23 | -.77 | .444 |
| % urban popul. in state (*γ_01_*) | -.004 | .004 | 60.72 | -.97 | .337 |
| % rural popul. in state (*γ_02_*) | -.006 | .006 | 58.06 | -1.02 | .312 |
| *Random Effects*  Intercept (*u*_0j_) | *Estimate*  .005 | *Std. Error*  .004 |  | *Wald Z*  1.35 | *p*  .176 |
| Residual (*ε*_ij_) | .373 | .011 |  | 33.05 | < .001 |
| **Model 6: Trust in family members** | | |  |  |  |
| *Fixed Effects* | *Estimate* | *Std. Error* | *df* | *t* | *p* |
| Intercept (*γ_00_*) | .859 | .382 | 68.75 | 2.25 | .028 |
| Information on phones (*γ_10_*) | .008 | .007 | 2257.70 | 1.05 | .295 |
| % urban popul. in state (*γ_01_*) | -.004 | .004 | 66.04 | -1.11 | .269 |
| % rural popul. in state (*γ_02_*) | -.008 | .005 | 63.21 | -1.51 | .136 |
| *Random Effects*  Intercept (*u*_0j_) | *Estimate*  .005 | *Std. Error*  .004 |  | *Wald Z*  1.47 | *p*  .142 |
| Residual (*ε*_ij_) | .358 | .011 |  | 33.16 | < .001 |

*Notes*. For all models, we used maximum likelihood estimation with unstructured covariance matrix on SPSS 21.

Generalized model equation: *Trust_ij_*=* γ_00_ + γ_10_Info-on-phone_ij_ + γ_01_Urban_j_ + γ_02_Rural_j_ + u_0j_ + ε_ij_*

*i = person level; j = state level*
